# Utilising Group-Size and Home-Range Characteristics of Free-Roaming Dogs (FRD) to Guide Mass Vaccination Campaigns against Rabies in India

**DOI:** 10.3390/vaccines7040136

**Published:** 2019-09-30

**Authors:** Harish Kumar Tiwari, Mieghan Bruce, Mark O’Dea, Ian D Robertson

**Affiliations:** 1 School of Veterinary Medicine, Murdoch University, 90 South Street, Perth, WA 6150, Australia; mieghan.bruce@murdoch.edu.au (M.B.); i.robertson@murdoch.edu.au (I.D.R.); 2 Ausvet, 5 Shuffrey Street, Fremantle, Perth, WA 6160, Australia; 3 China-Australia Joint Research and Training Center for Veterinary Epidemiology, Huazhong Agricultural University, Wuhan 430070, China

**Keywords:** mass vaccination, rabies, free roaming dogs, home range, group size

## Abstract

Adequate vaccination coverage of free roaming dogs (FRD) against canine rabies is not achieved primarily due to difficulties in administering parenteral vaccinations to this population. One factor associated with this difficulty is the tendency of FRD to form groups, which increases their aggressive behavior, resulting in a significant risk of dog-bites for the vaccinators. This study investigated factors that influenced FRD forming groups and their home-ranges, using data obtained from photographic capture-recapture/sight-resight surveys conducted in rural Shirsuphal (584 sightings) and urban Panchkula (3208 sightings), India. In the rural site, older dogs (OR 0.5, 95% CI 0.2–0.9, *p* = 0.03) and FRD sighted within 20 m of garbage sites (OR 0.6, 95% CI 0.4–0.9, *p* = 0.02) were less likely to be in groups. The number of dogs sighted with an FRD decreased with increased resight-probability of that dog (β = −1.0, *p* < 0.001). The rural FRD with smaller home-ranges were more likely to be sighted alone (OR 2.3, 95% CI 1.0–95, *p* = 0.04) than those with larger home-ranges. In the urban site, females (OR 1.3, 95% CI 1.1–1.5, *p* = 0.002) and older dogs (OR 1.5, 95% CI 1.1–2.1, *p* = 0.07) were more likely to be found in groups, and groups of dogs were more likely to be seen within 20 m of garbage sites (OR 1.7, 95% CI 1.5–2.0, *p* < 0.001). The distribution of urban FRD sighted alone, in pairs, triads, and in packs of ≥4 dogs were not random in the administrative (*p* = 0.02), and the two industrial (*p* = 0.03 & 0.01) survey tracks of the urban site, implying stable groups. The resighting probability of a dog (β = 0.3, *p* < 0.0001) and presence of garbage within 20 m (β = 0.2, *p* < 0.0001) in the urban site increased the likelihood of sighting a FRD with other dogs. It is concluded that data on the resighting probability, presence of garbage points, and home-ranges can be utilised to guide the selection of parenteral or oral rabies vaccination to achieve a population vaccination coverage of 70% to break the transmission cycle of rabies virus in FRD in India.

## 1. Introduction

The high incidence of dog-bite related rabies in India is attributed to the overwhelming presence of free-roaming dogs (FRD) [1]. The FRD population can contain two categories of dogs: those that depend on human settlements for food and shelter, and those that are bereft of any human association and are often classified as feral dogs [2]. The behavioural traits and demography of FRD are influenced by the socio-cultural and economic features of the human societies they are connected to [3], and their location is influenced by the availability of food and shelter, making them potentially responsible for the spread of zoonotic diseases [4,5]. In particular, the territoriality and movement of FRD greatly influences rabies transmission, and its spread can be modelled on the contact rates between FRD, which in turn depends upon their grouping behaviour and home-ranges [6].

The home-range of a free ranging animal is defined as the space it commonly uses for normal activities, such as foraging, hunting, and whelping [7,8]. The availability of food and shelter, and hence the home-range of an FRD, is strongly influenced by the attitudes of the human population towards them [9]. Free roaming dogs are known to display altered social behaviour and travel further for activities such as mating [10,11,12] and are also known to take isolated forays into neighbouring villages/localities due to human community or climatic events [13], which increases the likelihood of becoming infected with rabies virus due to potential contact with a larger population of dogs or other potentially infected wildlife. Although studies on the behaviour and home-ranges of FRD have been conducted elsewhere [4,10,12,14,15] and in urban areas in India [11,16,17,18,19], there is a lack of such information regarding FRD in rural areas of India.

A major impediment against successful immunisation programmes against canine rabies is the failure to achieve the prerequisite vaccination coverage [20], primarily due to difficulties in catching FRD and their frequent inaccessibility [21,22]. Nets have been used to capture and restrain dogs in India during mass parenteral immunisation campaigns against rabies [23]; however, there are significant occupational risks for those involved. In spite of being expensive, Oral Rabies Vaccination (ORV) can be judiciously used in areas where poor catchability/accessibility of FRD precludes achieving 70% population immunisation coverage against rabies by parenteral inoculation [24,25].

The planning and implementation of successful mass vaccination campaigns against rabies requires an understanding of the demographic characteristics of the FRD population and their propensity to form social groups [26,27,28]. Investigation of the determinants that promote FRD to be sighted together, along with their home-ranges, can help design effective vaccination efforts including both parenteral and oral vaccination programmes. In the present study, we explore whether mathematical interpretation of FRD grouping behaviour and home-ranges can inform decision-making of the most effective intervention or combination of interventions to adopt against rabies. This study was undertaken in urban and rural locations of western and northern India to (1) investigate the predictors of group forming behaviours of FRD; (2) evaluate the home-ranges of frequently sighted FRD and their determinants, and (3) compare and contrast the home-ranges and tendency to form groups between urban and rural FRD to make an informed decision for adopting suitable interventions against rabies in these localities. 

## 2. Materials and Methods

### 2.1. Study Area and Field Methodology

This study utilised the data generated during our photographic sight-resight studies on enumeration of FRD in a rural location of Shirshupal village, Maharashtra state [13], and 15 urban sectors administered by the Municipal Corporation, Panchkula, Haryana state, India [29]. The photographic sight-resight technique for FRD, where photographs of dogs are used to identify and describe dogs at the time of their sighting along with their GPS location, has been used in a number of studies [13,29,30,31,32]. In this study the gender, age, and body condition of the FRD were assessed through visual evaluation. 

### 2.2. Analysing Distribution of Different Sized FRD Groups across Time and Space

An individual FRD was classified as being sighted alone (solitary) or in a group (presence of another dog within 10 m) [14]. Groups were further categorised as a pair, triad, or a pack (≥four dogs).

To analyse the distribution of the different-sized groups, we developed a model for random distribution and tested it against the data obtained from the rural and urban surveys. Assuming that X_*i*_ is the group-size (solitary, pair, triad or pack) of the FRD, Oi is the frequency with which the group-size X_*i*_ is sighted during the survey, and *P*(*x*) is the probability of sighting the group-size X_*i*_, then, if FRD are randomly distributed in the village, the probability distribution, *P*(*x*) follows a Poisson distribution, where the composition of the FRD group is independent of any particular dog in the group. As the distribution of the response variable (observed group-size of the FRD) should ideally follow a Zero-truncated Poisson distribution (ZTPD) with no chance of the frequency being 0 (every sighting comprises the detection of at least one dog), we tested the observed frequencies with expected frequencies for goodness of fit of the data to a ZTPD using a χ2 test [14,16,33].

Mathematically this equates to the following formula:

(1) $$P\left(x\right)=\frac{e^{-\lambda \;}\lambda^{x\;}\;}{x!}\times \frac{1}{1-e^{-\lambda }}$$

where *λ* is obtained from the mean of the distribution μ, and is equal to *λ* ÷ (1 − e^−*λ*^) (obtained from Molina’s table for the Poisson function) [34]. If $E_{𝒾}$ is the expected frequency of group-size, $X_{𝒾}$, then $E_{𝒾\;}=\;N\times P\;\left(X_{𝒾}\right)$, where N is the total number of dogs sighted each time and is equal to $\sum_{i}E_{i\;}O_{i}$. The mean of the distribution (μ) was calculated by dividing N by the number of sightings of different sized groups and the values of *λ* for the observed and expected frequencies for the Poisson distribution were obtained from Molina’s table. In other words, if the distribution of different group-sizes were random in space and time, we could infer that the FRD sighted together were together only by chance and not due to the existence of stable social associations [16,33].

The distributions of the different group-sizes in rural Shirsuphal and urban Panchkula were tested for goodness of fit to the ZTPD-process for the temporal and spatial data.

### 2.3. Analyses of Factors that Influence the Number of FRD Sighted Together

Intrinsic (age, body condition, gender, de-sexing) and extrinsic (ambient temperature, humidity, wind velocity, and the dog’s proximity to garbage) influences on the tendencies of FRD to form groups were analysed on the basis of the sightings of the individually identifiable dogs during a series of photographic sight-resight surveys. These variables were recorded on the basis of the visual appreciation of the sighted dogs (age—pup/young/adult/old; body condition—good/poor/ fair; gender—male/female; and de-sexing—yes/no). The gender of some dogs could not be confirmed, and these were removed from the final analyses. The regression analyses of various predictors (categorical—age, gender, within 20 m of a garbage site/bin, body condition; and numerical—ambient temperature, humidity, wind velocity on the day of the survey, and resight probability of an FRD) on the numerical response variable (actual number of FRD sighted together) were performed using generalised linear mixed model analyses [35]. Initially, the regression of the response variable (number of FRD sighted together) on the predictors were tested with univariable models. A saturated multivariable model was then constructed by including all variables yielding a *p*-value < 0.25 on the univariable analyses. A reduced subset model was subsequently achieved by using a backward elimination approach with predictors excluded based on their *p*-values. The predictors were tested for confounding by excluding them from the final model and observing the change in the coefficient values of the fixed effects. If the intercept changed by more than 20% and the AIC (Akaike Information Criterion) score decreased by more than two points, the predictor was retained in the model, otherwise it was excluded. 

### 2.4. Analysis of Home-Range

All dogs that were sighted on more than four occasions were selected for determining home-ranges using the Maximum Convex Polygon (MCP) method. A logistic regression model was developed to investigate the determinants that affect the home-ranges, and for this purpose two variables were dichotomised at the median score—the response variable, home-range area, (≤0.11 ha and >0.11 ha in rural Shirsuphal, and ≤1.07 ha and >1.07 ha in urban Panchkula); and the predictor variable, probability of being sighted alone (≤0.42 and >0.42 in rural Shirsuphal ; and ≤0.2 and >0.2 in urban Panchkula). Initially, the putative variables were tested for association with the dependent variable by a χ2 or Fisher’s exact test (univariable analyses) and only those variables with *p*-values of <0.25 were selected for inclusion in the final multivariable logistic regression model through backward elimination.

### 2.5. Data Entry, Storage, and Cleaning and Analyses

All population survey data were entered and organised in Microsoft Excel (2007). The tracks and waypoints recorded on the GPS were converted into Excel files using the online tool, www.mygeodata [36]. The data were then cleaned by removing unwanted data records, except for individual identity and location coordinates, and converted to CSV files for use in R programming environment [36]. 

The R package ‘glmmTMB’ was used to construct the regression model of factors that influenced the number of FRD sighted together. This package caters for the random effects (individual heterogeneity, survey-track and survey occasion) for truncated Poisson distributions with the predictors as fixed effects and the outcome as a numerical response [37]. The validity of the model was tested using the ‘DHARMa’ package by visual appreciation of the distribution of the simulated residuals (number of simulations = 10,000) using Q-Q plots, along with checking the homoscedasticity by plotting fitted model predicted values vs. standardised residuals [38]. The normality of the residuals distribution was verified by using a one-sample Kolmogorov-Smirnov (KS) test. The R package ‘adehabitatHR’ [8] was used to estimate home-ranges. A logistic regression model was constructed in R and the validity of the final logistic model was tested with ‘general hoslem’ [39] and an ANOVA (LRT). All statistical analyses were performed using R version 3.4.4 [40].

## 3. Results

### 3.1. Sightings of the FRD in Rural and Urban Settings as Solitary or in Groups

There were 584 and 3208 sightings of FRD, with a mean resighting probability of 0.51 and 0.62 in the rural and urban settings, respectively. The details of the number of FRD sighted alone or in groups are presented in Table 1. The FRD in urban Panchkula were more likely to be sighted in groups (OR 2.7, *p* < 0.001) than in rural Shirsuphal. 

No temporal variation was observed in the number of solitaries, pairs, triads or packs (≥4) of dogs sighted across the seven survey days in rural Shirsuphal (χ2 = 14.35, df =18, *p* = 0.7) and the distribution of the different sized groups of FRD followed a ZTPD (*p* = 0.09). The temporal frequency distribution of the count of the different sized groups along all survey tracks in urban Panchkula across five days of survey followed a ZTPD, except for the perimeter and the administrative survey tracks (χ2 = 25.4, df = 12, *p* = 0.01 and χ2 = 22.5, df = 12, *p* = 0.03, respectively). 

The spatial distribution of the solitaries, pairs, triads or packs (≥4) of FRD sighted across four major survey-tracks (A1, A2, B1, B2) in rural Shirsuphal varied significantly (χ2 = 25.42, df = 9, *p*-value = 0.0005), with the pattern for two tracks (A1 and B1) not following a ZTPD. The distribution of different group sizes across the survey tracks in urban Panchkula also differed significantly (χ2 = 119.97, df = 26, *p* < 0.0001), but no significant difference was observed within the tracks of similar settings (residential: χ2 = 20.7, df = 15, *p* = 0.14; industrial: χ2 = 0.9, df = 3, *p* = 0.8; part-residential part-public facilities: χ2 = 5.5, df = 6, *p* = 0.5; and sectors comprising unplanned colonies/villages/slums: χ2 = 5.9, df = 3, *p* = 0.11). However, the frequencies of the counts of different sized groups for survey tracks in Parts I (χ2 = 8.6, df = 3, *p* = 0.03) and II (χ2 = 10.9, df = 3, *p* = 0.01) of the industrial sectors, the administrative sector (χ2 = 10.1, df = 3, *p* = 0.02) and the perimeter track (χ2 = 10.9, df = 3, *p* = 0.01) did not follow a ZTPD. 

Female FRD were more likely to be sighted with other dogs than male FRD in rural (OR 1.8, 95% CI 1.3–2.7, *p* = 0.0008) and urban settings (OR 1.3, 95% CI 1.1–1.5, *p* = 0.002). Old FRD were more likely to be sighted with other dogs in urban Panchkula (OR 1.5, 95% CI 1.1–2.1 *p* = 0.007) than adult dogs, whereas in rural Shirsuphal, old FRD were less likely to be sighted with other dogs (OR 0.5, 95% CI 0.2–0.9, *p* = 0.03). Similarly, in urban Panchkula, FRD were more likely to be sighted in a group (≥2 dogs) within 20 m of garbage sites than alone (OR 1.7, 95% CI 1.5–2.0, *p* < 0.001). In contrast, in rural Shirsuphal, FRD were less likely to be sighted in a group within 20 m of garbage sites (OR 0.6, 95% CI 0.4–0.9, *p* = 0.02). The odds for different categories of FRD (sex, age, body condition, de-sexed, and within 20 m of a garbage site) being sighted alone or together with other dogs are presented in Table 2.

### 3.2. Determinants of Number of FRD Sighted Together or Alone

The univariable analyses of factors that may influence the number of FRD which were sighted together in rural and urban settings are presented in Table 3. In the final multivariable model for dogs in rural settings, resight probability (β = −1.0, SE = 0.2, *p* < 0.001), fair body condition (β = −0.3, SE = 0.1, *p* = 0.008) and sighted within 20 m of a garbage point (β = 0.2, SE = 0.1, *p* = 0.03) were found to have a significant effect on the actual number of FRD sighted together (Table 4). The model was found to fit the data well, with the residuals on 10,000 simulations being normally distributed (Kolmogorov-Smirnov test, D = 0.05, *p* = 0.15).

The final multivariable model for urban settings determined that the resighting probability (β = 0.3, SE = 0.06, *p* < 0.0001) and the sighting of a FRD within 20 m of a garbage point (β = 0.2, SE = 0.03, *p* < 0.0001) positively influenced the actual number of FRD sighted together (Table 5). A negative influence was observed for adult (β = −0.2, SE = 0.07, *p* = 0.03) and old (β = −0.3, SE = 0.09, *p* = 0.02) FRD. De-sexed animals were less likely to be sighted with other FRD than entire dogs (β = −0.07, SE = 0.03, *p* = 0.05). The influence of temperature and humidity was significant but inconsequential considering the per unit change on the number of FRD sighted together. The model could not be evaluated for goodness of fit by testing the simulated residuals as it failed to converge after 10,000 iterations. However, the model did fit the data well when random subsets (150 to 250 observations) of the data were tested for normal distribution of the residuals.

### 3.3. Home-Range of FRD and its Determinants

The average home-ranges of dogs with four or more sightings were similar (*p* = 0.3); rural FRD (*n* = 29) had a median home-range of 2.8 ha (minimum = 0.0021 ha, maximum = 50.48 ha), and urban FRD (*n* = 74) had a median home-range of 3.7 ha (minimum 0.0065 ha, maximum = 40.6 ha) (Supplementary Table S1). The associations between the home-ranges and the predictor variables for rural Shirsuphal and urban Panchkula are presented in Table 6. Although the variable ‘sex’ yielded a *p*-value of 0.26, it was included for building the multivariable regression model. In the final multivariable logistic regression model for the factors influencing the dichotomised median home-range (>0.42, ≤ 0.42 ha) of FRD in rural Shirsuphal, gender and the probability of being sighted alone were retained (Table 7). The Hosmer-Lemeshow test for goodness of fit could not be applied to evaluate the model due to the small number of observations, but it was found to be weakly stable on the ANOVA (LRT) test (*p* = 0.02). The association between the two predictors in the final model was not significant (*p* = 0.11). A final multivariable logistic regression model for the influences on the dichotomised median home-range of urban FRD could not be produced, as only one factor (body condition) significantly influenced home-range size.

## 4. Discussion

In the current study, the likelihood of sighting a dog in a group was higher in the urban study site than in the rural site. However, irrespective of the study site, FRD were sighted in groups close to a food resource, similar to that reported by Berman and Dunbar [14] in the city of Berkeley, CA, USA. The proportion of FRD sighted in groups in urban Panchkula (73.7%) was higher than in Berkeley (17.8%) because in Panchkula, FRD were observed to group in public places, such as outside of temples and community markets where they potentially received food from visitors and had access to shelter (shaded areas), which may not have been the case in the Berkeley study. The difference in the proportion of FRD forming groups in rural and urban sites in our study is possibly due to a lack of organised garbage management in rural Shirsuphal that results in edible litter being thrown indiscriminately throughout the location, including lanes (roadways), reducing the need for dogs to congregate at specific garbage refuse points. In urban Baltimore, MD, USA, a higher proportion of FRD were sighted in groups, which was believed to be due to the presence of garbage in the alleyways [14,15]. In contrast, in urban Panchkula, edible waste was usually deposited at the assigned waste disposal points, such as garbage bins and dumps, although these were frequently overflowing with litter, accounting for the likely congregation of FRD at these locations.

The observations regarding the categories (gender, age, body condition, proximity to garbage, and de-sexing) likely to be sighted in groups were consistent for both rural Shirsuphal and urban Panchkula, except for the proximity to garbage points (Table 2). 

The temporal distribution of the groups across the days of the sight-resight surveys was random in rural Shirsuphal and for most of the survey tracks in urban Panchkula. However, the spatial frequency distribution of various sized groups in three of the tracks in rural Shirsuphal was not random, implying the tendency of FRD to form stable groups at these sites. A possible explanation for stable groups in rural Shirsuphal could have been the presence of lactating bitches (13%) and bitches in oestrous (6%) on these tracks [10,35,41,42], but the univariable analyses of the influence of gender over group sizes of FRD did not show any significant difference between the group forming tendency for male and female FRD (Table 3). The FRD groupings in rural Shirsuphal can be attributed to individual preferences of the rural FRD to stay together rather than the need to form stable groups for hunting/sourcing food [16]. This observation is supported by Boitani, Ciucci [10], who speculated that FRD have a lower tendency to form stable social groups in locations where food is readily available. Nevertheless, it is possible that factors other than lactation and oestrous, such as level of human interaction, may be important and further studies are warranted in similar locations to identify these factors which restrict group formation for rural FRD. 

In urban Panchkula, the evidence of temporally and spatially stable groups (*p* = 0.01 and *p* = 0.02, respectively) in the perimeter survey track in urban Panchkula is not unexpected as FRD are known to form temporary groups while transiting between their usual locations [14], such as between adjacent sectors in this study. However, the distribution of the different sized groups also did not follow a ZTPD in the industrial (*p* = 0.03 and *p* = 0.01 for Part I and Part II, respectively) and administrative sectors (*p* = 0.02), which suggests the presence of stable hierarchical social groups, similar to that reported in some international studies [43,44] and elsewhere in India [11]. Daniels and Bekoff [40], who similarly found that the group sizes of FRD in Mexico did not follow a ZTPD, believed that the dog-ownership practices of the resident community in the area influences the level of social organisation of FRD. The authors claimed that the dogs that were cared for by owners did not form groups with conspecifics, and thus, the pattern of their social organisation would differ depending upon their response to the amount and provision of food resources. In our study, although the dogs were not owned in rural Shirsuphal, they were provided food by the rural residents [45], hence limiting the tendency of rural FRD to form groups.

FRD that had been neutered were less likely to be part of a group than entire FRDs (β = −0.08, *p* = 0.05) (Table 5). This finding is supported by the presence of stable groups in the industrial and administrative sectors in urban Panchkula where more dogs had not been neutered (Section 3.1). We found that the odds of sighting a de-sexed FRD in industrial (OR 0.7, *p*-value = 0.01) and administrative (OR 0.2, *p*-value <0.001) survey tracks was lower compared to residential sectors. Interestingly, this finding is similar to the study in the city of Petrozavodsk, Russia, where the number of stray dogs forming groups in industrial zones was higher than in residential areas where most dogs were solitary [42]. This was believed to be linked with the presence of secluded breeding dens and fewer instances of human interference restricting the formation of packs in these zones. Another plausible explanation of sighting fewer de-sexed FRD could be that they are harder to catch, being frequently sighted in groups. The inaccessibility for parenteral vaccination is pronounced in stable FRD groups, possibly due to their enhanced agonistic behaviour toward humans and the tendency to avoid humans when in groups [46]. An anecdotal admission by the dog catchers of Panchkula Municipal Corporation regarding the heightened risk of dog-bites in the industrial and administrative sectors also corroborated the higher likelihood of stable social groups in these sectors. 

The average home-ranges (2.8 ha and 3.7 ha for rural and urban settings, respectively) are comparable to those reported in USA, Australia, Russia, and India for urban FRD [14,18,42], but were much lower than that reported in Chile (65 ha) [47] and Aboriginal and Torres Strait Islander communities in Northern Australia (40–104 ha) [4]. Although our study limited the inclusion of FRD to those with a high resight probability (≥0.7), most of these individuals were sighted alone. In rural Shirsuphal, dogs that were most likely sighted in a group were likely to have larger home-ranges (OR 2.3, 95% CI 1.0–95.0, *p* = 0.04, Table 7) than those mostly sighted alone. This finding, in conjunction with that of the influence of resight probability on grouping tendency (β = −1.0, SE = 0.2, *p* < 0.001, Table 4), imply that rural FRD find the resources (food, shelter) for survival within a small area and hence do not need to wander. More than half of the rural FRD (59%) for whom the home-range calculation was possible were never seen in the vicinity of garbage points, and the remaining were sighted near garbage (<20 m) only once or twice during the survey period. This strongly supports the belief that the dogs with smaller home-ranges have higher human affinity in rural Shirsuphal and thus may be more amenable for administering parenteral vaccination against rabies.

Notwithstanding the comparable home-ranges in rural and urban settings, the grouping tendency of FRD in urban Panchkula needs to be examined in relation to the resight probability. The resight probability was higher for smaller sized groups in rural Shirsuphal (β = −1.0, SE = 0.2, *p*-value< 0.001), whilst in urban Panchkula resight probability increased with group size (β = 0.3, SE = 0.06, *p* < 0.0001) indicating that FRD in the urban setting are likely to be sighted more when they are in groups and around a food resource (garbage) (Table 1 and Table 2). Even though we failed to produce a multivariable model that could investigate the influence of resight probability on home-ranges on FRD in urban Panchkula, resight probability is positively related to the tendency of urban dogs to form groups (Table 5).

These observations can be corroborated with other findings where stray/semi-owned dogs remain in proximity to people who provide food to them, even when those people do not claim ownership of the dogs [47,48,49]. Consequently, we conclude that FRD in rural Shirsuphal are more accepting of human proximity than those in urban Panchkula. Human influence over the home-ranges of FRD was also demonstrated by Ivanter and Sedova [42] and Boitani, Ciucci [10], who reported smaller home-ranges for dogs in areas close to humans. This finding, in conjunction with that of group-size, implies that if a photographic capture-recapture survey reveals solitary dogs with high resighting probability and small home-ranges, then more dogs will be accessible for parenteral vaccination against rabies. We speculate this may largely be true for rural India, although it is recommended that more studies are conducted to support this recommendation. In contrast, ORV should be adopted in areas where FRD are more likely to be sighted in groups. Gibson, Yale [50] demonstrated in Goa, India that Oral Bait Handout (OBH) vaccines are a viable option for the FRD that may be accustomed to human presence but resist retraining them for vaccine inoculation. Consequently, based on our findings of urban Panchkula we also suggest that, irrespective of the measure of the home-ranges, a high resight probability of FRD is indicative of a higher proportion of groups and hence ORV should be implemented to achieve adequate herd immunity. Oral rabies vaccination has been recommended where catchability of FRD for parenteral immunisation is difficult [51]. Although ORV has the disadvantages of high cost and the need for strict supervision, its potential use to augment parenteral inoculation to achieve 70% coverage in FRD has been advocated [25]. Once the required number to vaccinate 70% of the total population is estimated through a reliable enumeration technique [13], it is recommended that the data obtained through such enumeration surveys can be used to model the grouping tendencies of the FRD. Based on this grouping tendency, the most effective method(s) can be selected achieve adequate mass immunisation against canine rabies in India. 

There were some limitations encountered during the current investigation. Firstly, we used GPS fixes of the FRD at pre-set survey times in the multiple sight-resight survey sessions. The use of GPS collars would have given more frequent GPS fixes and hence a better assessment of home range, although this would have been offset by a smaller sample size due to significantly higher costs associated with this methodology. However, the need for multiple survey sessions can be off-set by using GPS-collars on randomly selected FRD from different locations of the study sites to allow estimation of average home ranges. Second, only one rural village was included in this study, and more villages should be studied to improve the robustness of the findings. Third, we were unable to develop a multivariable logistic model for factors influencing the home-range of the urban FRD, and consequently data on other influences need to be collected. Finally, bias due to observer fatigue toward the end of multiple sight-resight surveys cannot be completely ruled out [52]. In spite of these limitations, this investigation linked the behaviour of FRD and home-ranges with their accessibility for future vaccination programmes to achieve the necessary herd immunity to control canine rabies. We recommend more studies are conducted at different urban and rural locations in India to model the group-size of FRD, based upon easily measurable predictors, including home-ranges to make informed decisions on the FRD mass vaccination approach to adopt to control rabies.

## 5. Ethical Approval

Ethics approval for the observation of the FRD in the rural and urban areas was obtained from the Ashoka Trust for Research in Ecology and the Environment (ATREE) (AAEC/101/2016). 

## Figures and Tables

**Table 1:** The number and likelihood of free roaming dogs (FRD) being sighted alone or with other dogs during the photographic sight-resight survey carried out in Shirsuphal (rural) and selected sectors of Panchkula (urban), India.

Variable	Number of FRD Sighted; *n* (%)		OR (95% CI)
	Rural	Urban	
Total Sighted	584 (100)	3208 (100)	
Sighted alone	288 (49)	844 (26)	1.0
Sighted with other dogs (≥2)	296 (51)	2364 (74)	2.7 (2.3–3.3) *
Sighted alone	288 (49)	844 (26)	1.0
Sighted in a pair	130 (22)	759 (24)	2.0 (1.6–2.5) *
Sighted in a triad	67 (12)	580 (18)	2.9 (2.2–3.9) *
Sighted in a pack (≥4 dogs)	99 (17)	1025 (32)	3.5 (2.7–4.5) *
Mean resight probability	0.51 (0.14–1.0)	0.62 (0.2–1.0)	
Mean group size ^†^	1.98	3.03	

* *p*-value < 0.001; ^†^ FRD sighted alone were included as group size 1 in the calculation.

**Table 2:** The odds of various categories of FRD being sighted in groups (≥ 2) in rural (Shirsuphal) and urban (Panchkula) settings.

Factors	RURAL (Shirsuphal)				URBAN (Panchkula)			
	Number Sighted	Number Sighted in a Group * (%)	OR (95% CI)	*p*–Value	Number Sighted	Number Sighted in a Group * (%)	OR (95% CI)	*p*-Value
Gender					Gender			
Male	415	192 (46)	1.0	–	1791	1282 (72)	1.0	–
Female	169	104 (62)	1.8 (1.3–2.7)	0.0008	1417	1082 (76)	1.3 (1.1–1.5)	0.002
Age ^†^					Age ^†^			
Pup	30	16 (53)	1.0 (0.5–2.3)	0.88	118	99 (84)	1.9 (1.2–3.2)	0.01
Young	100	51 (51)	1.0 (0.6–1.5)	0.86	328	257 (78)	1.3 (1.0–1.7)	0.04
Adult	416	216 (52)	1.0	–	2584	1894 (73)	1.0	–
Old	38	13 (34)	0.5 (0.2–0.9)	0.03	178	114 (64)	1.5 (1.1–2.1)	0.007
Body condition					Body condition			
Good	361	210 (58)	1.0	–	2212	1635 (74)	1.0	–
Fair	199	119 (60)	1.06 (0.7–1.5)	0.7	521	376 (72)	0.9 (0.7–1.1)	0.4
Poor	24	18 (75)	2.1 (0.8–6.0)	0.1	475	353 (74)	1.02 (0.8–1.3)	0.8
Proximity to garbage (within 20 m)					Proximity to garbage (within 20 m)			
Yes	140	50 (36)	1.0	–	1571	1241 (79)	1.0	–
No	444	206 (46)	0.6 (0.4–0.9)	0.02	1637	1124 (69)	1.7 (1.5–2.0)	<0.0001
De-sexed					De-sexed			
Yes	0	0			849	595 (70)	1.0	–
No	584	296 (51)			2359	1769 (75)	1.3 (1.1–1.5)	0.005

* Group refers to ≥ 2 dogs together. ^†^ Age was assessed based on visual characteristics as Pup (≤6 months), Young (six months to 1 year), Adult (>1–7 years), and Old (≥7 years).

**Table 3:** The coefficients, standard error and *p*-values of the univariable generalised linear mixed models for the regression of predictor variables on the actual group size of FRD * in rural Shirsuphal ^#^ and urban Panchkula ^†^ in India.

	RURAL (Shirshuphal)			URBAN (Panchkula)		
Factors	Coefficient (β)	SE	*p*-Value	Coefficient (β)	SE	*p*-Value
Resight Probability	−0.9	0.2	0.0004	0.3	0.07	<0.001
Temperature	0.02	0.02	0.3	−0.02	0.003	<0.001
Humidity	−0.004	0.004	0.3	0.006	0.001	<0.001
Wind velocity	0.01	0.008	0.2	−0.03	0.01	0.003
Gender						
Female	1.0	–	–	1.0		
Male	−0.14	0.14	0.3	−0.03	0.03	0.5
Age^@^						
Pup	1.0	–	–	1.0	–	–
Young	−0.18	0.33	0.6	−0.01	0.1	0.9
Adult	−0.04	0.30	0.9	−0.2	0.1	0.01
Old	−0.28	0.41	0.5	−0.3	0.1	0.03
Body condition						
Good	1.0	–	–	1.0	–	–
Fair	−0.3	0.14	0.02	−0.05	0.04	0.3
Poor	−0.8	0.4	0.05	0.006	−0.05	0.9
Proximity to garbage site (within 20 m)						
No	1.0	–	–	1.0		
Yes	0.2	0.1	0.03	0.2	0.03	<0.001
De-sexed	NA	NA	NA			
No				1.0	–	–
Yes				−0.08	0.04	0.03

* Size of groups in rural Shirsuphal ranged between one (single dog) to five FRD per group and between 1–12 FRD in urban Panchkula; ^#^ Variance from the random effects due to repeat identity of FRD on successive surveys and day of the survey was negligible (<0.05 and <1.5x 10–9, respectively); ^†^ Variance ± SD from the random effects due to repeat identity of FRD on successive surveys and survey-tracks was 0.2 ± 0.4 and, 0.04 ± 0.2, respectively for all variables; @Age was assessed based on visual characteristics as Pup (≤6 months), Young (six months to 1 year), Adult (>1–7 years), and Old (7 years); NA–Not Applicable.

**Table 4:** Final multivariable model of predictors that significantly influenced the number of FRD sighted together (1–5) in a rural setting *.

Factors	Coefficient (β)	SE	*p*-Value
Constant	0.6	0.1	
Resight Probability	−1.0	0.2	<0.001
Wind velocity (km/hour)	0.02	0.01	0.07 ^†^
Body condition			
Good	1.0	–	–
Fair	−0.3	0.1	0.008
Poor	−0.7	0.4	0.06
Proximity to garbage site (≤20 m)			
No	1.0		–
Yes	0.2	0.1	0.03

* Variance from the random effect due to individual heterogeneity = 0.4 ± 0.6; day of survey = 0.0001 ± 0.01; ^†^ removal of the factor enhanced the Akaike Information Criteria (AIC) to 1509.4 of the model, consequently a more stable model was selected by including this factor with AIC of 1507.9.

**Table 5:** Final multivariable model of predictors that significantly influenced the number of FRD sighted together (1 to 12) in an urban setting *.

Factors	Coefficient (β)	SE	*p*-Valve
Constant	−0.7	0.4	
Resight Probability	0.3	0.06	<0.0001
Temperature (°C)	0.02	0.01	0.03
Humidity (%)	0.01	0.003	<0.0001
Age ^@^			
Pup	1.0	–	–
Young	−0.01	0.08	0.9
Adult	−0.2	0.07	0.03
Old	−0.3	0.09	0.02
De-sexed?			
No	1.0	–	–
Yes	−0.07	0.03	0.05 ^†^
Proximity to garbage site (≤20 m)			
No	1.0	–	–
Yes	0.2	0.03	<0.0001

* Variance from the random effect due to individual heterogeneity = 0.2 ± 0.4; survey-track = 0.03 ± 0.2; ^†^ removal of the factor enhanced the AIC (11569.8) of the model, consequently a more stable model was selected including this factor (AIC 11568.1); ^@^Age was assessed based on visual characteristics as Pup (≤6 months), Young (six months to 1 year), Adult (>1–7 years), and Old (≥7 years).

**Table 6:** Test of association between dichotomised home-range * and predictor variables for the rural (Shirsuphal) and urban (Panchkula) locations.

Variable	Rural *				Urban *			
	*N* = 29	*n* (%)	OR (95% CI)	*p*-Value	*N* = 74	*n* (%)	OR (95% CI)	*p*-Value
Gender								
Female	11	4 (36)	1.0	–	33	17 (52)	1.0	–
Male	18	11 (61)	2.6 (0.5–17.5)	0.26	41	21 (51)	1.0 (0.4–2.5)	0.9
Age ^†^								
Pup	1	1 (100)	–	–	4	3 (75)	1.0	–
Young	7	3 (43)	1.0	0.3	9	6 (67)	0.7 (0.01–14.3)	1
Adult	18	9 (50)	1.3 (0.2–7.7)	0.3	54	25 (46)	0.3 (0.005–3.9)	0.7
Old	3	2 (67)	2.7 (0.2–45.1)	0.5	7	4 (57)	0.5 (0.006–10.5)	0.5
Body condition								
Fair	10	4 (40)	1.0	–	19	4 (21)	1.0	–
Good	17	9 (53)	1.7 (0.4–8.2)	0.5	44	28 (63)	6.3 (1.6–30.9)	0.002
Poor	2	2 (100)	–	1	11	6 (54)	4.2 (0.7–30.8)	0.06
Probability of being sighted alone ^#^								
High	16	6 (37)	1.0	–	9	5 (56)	1.0	–
Low	13	9 (69)	3.5 (0.7–19.0)	0.13	65	33 (45)	1.2 (0.2–6.7)	0.9
Proximity to garbage ^@^								
No	17	9 (53)	1.0	–	25	11 (44)	1.0	–
Yes	12	6 (50)	0.9 (0.2–4.1)	0.99	49	27 (55)	1.5 (0.6–4.2)	0.36
De-sexed	NA	NA	NA	NA				
No					49	28 (57)	1.0	–
Yes					25	10 (40)	0.5 (0.2–1.3)	0.16

* Home-range dichotomised as ≤0.11 ha and >0.11 ha (the median home-range of the sampled population in Shirsuphal) and ≤1.07 ha and >1.07 ha (the median home-range of the sampled population in Panchkula); ^†^ Age was assessed based on visual characteristics as Pup (≤6 months), Young (six months to 1 year), Adult (>1–7 years), and Old (≥7 years). ^#^ the probability of a free-roaming dog sighted solitary was dichotomised as high or low (>0.42 or ≤0.42 for rural Shirsuphal, and >0.2 or ≤ 0.2 for urban Panchkula); ^@^ at least one sighting within 20 m of a garbage dump across the survey period.

**Table 7:** Final multivariable logistic regression model with regression coefficient values for predictors yielding significant *p*-values for dichotomised home-range area for free-roaming dogs in rural Shirsuphal, India.

Factors	*N* = 29	Coefficient (β)	SE	*p*-Value	OR (95% CI)
Constant		−2.2	1.2		
Probability of being sighted alone					
High (>0.42)	16	–	–		1.0
Low (≤0.42)	13	2.3	1.1	0.04	2.3 (1.0–95.0)
Gender					
Female	11	–		–	1.0
Male	18	2.1	1.2	0.07	8.1 (0.8–81.3)

Likelihood ratio test (LRT): Deviance 7.4, df = 2, *p*-value 0.02. * Home-range dichotomised as ≤0.11 ha and >0.11 ha (the median home-range of the sampled population in Shirsuphal).

## Supplementary Materials

The following are available online at https://www.mdpi.com/2076-393X/7/4/136/s1, Figure S1: Boxplots for the univariable analyses of regression of the various intrinsic and extrinsic factors on the group size of free roaming dogs (FRD) sighted during the enumeration survey in Shirsuphal village of western India in June 2016, Figure S2: Boxplots for the univariable analyses of regression of the various intrinsic factors on the group size of FRD sighted during the enumeration survey in Municipal Corporation Panchkula in northern India during September–October 2016, Figure S3: Boxplot graphics for the univariable analyses of regression of the various extrinsic factors on the group size of FRD sighted during the enumeration survey in rural and urban setting, Figure S4: Boxplots for the univariable analyses of regression of resight probability on the group size of FRD sighted during the enumeration survey in rural and urban settings, Figure S5: Boxplots for the univariable analyses of de-sexed status probability on the group size of FRD sighted during the enumeration surveys in the urban setting in Panchkula, Figure S6: Graphic representation of the odd ratios to compare the influence of various factors (probability of being sighted alone, gender, sighted within 20 m of garbage, if de-sexed, and body condition) on the home range of FRD sighted during the enumeration surveys in rural and urban settings through univariable analysis.
