# Supplementary material for: Utilising Group-Size and Home-Range Characteristics of Free-Roaming Dogs (FRD) to Guide Mass Vaccination Campaigns against Rabies in India
Source: Vaccines (Basel). 2019 Sep 30;7(4):136. doi: 10.3390/vaccines7040136 (PMC6963394; doi:10.3390/vaccines7040136)

**SUPPLEMENTARY FILE**

Supplementary Figure S1. Boxplots for the univariable analyses of regression of the various intrinsic and extrinsic factors on the group size of free roaming dogs (FRD) sighted during the enumeration survey in Shirsuphal village of western India in June 2016.


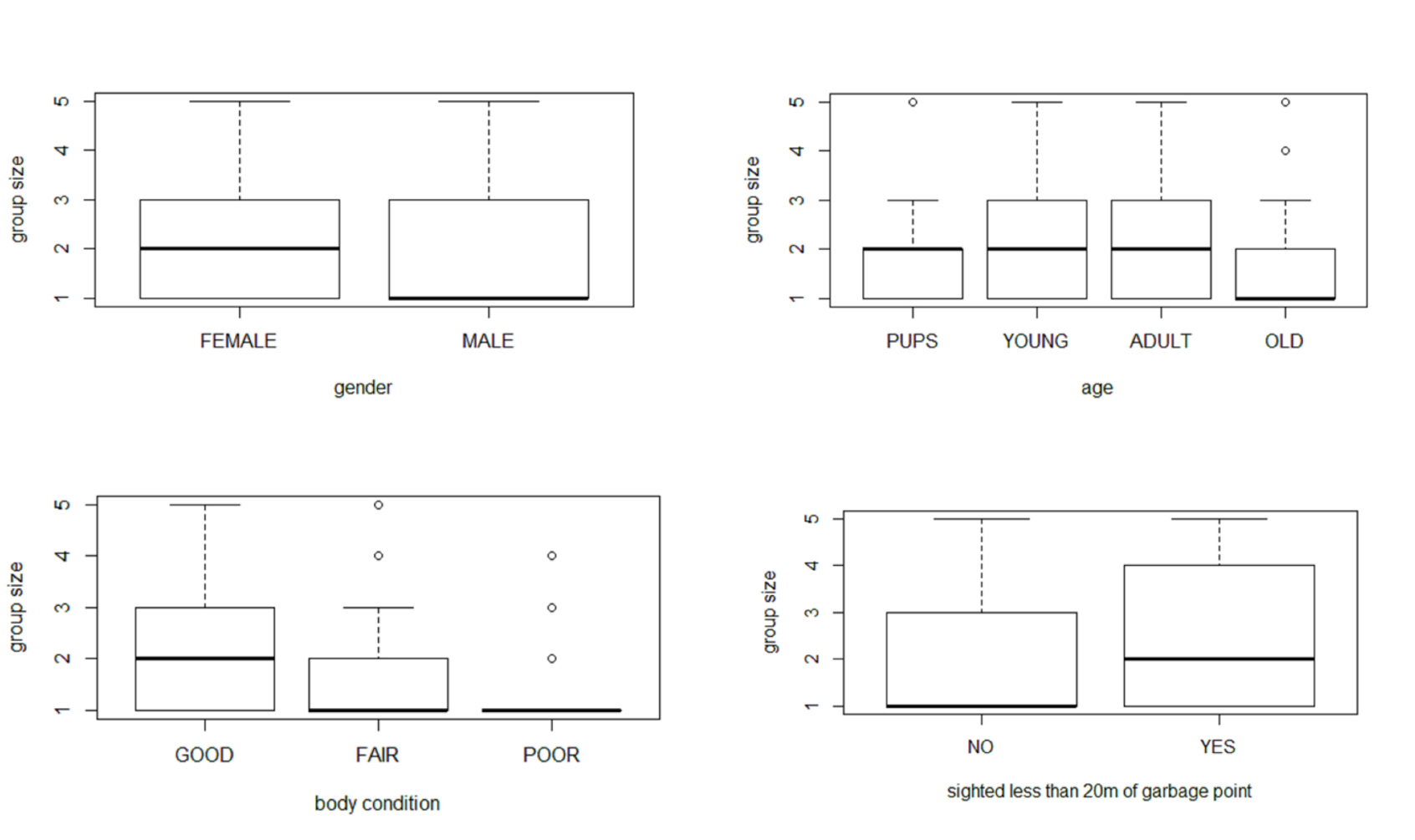


Supplementary Figure S2. Boxplots for the univariable analyses of regression of the various intrinsic factors on the group size of FRD sighted during the enumeration survey in Municipal Corporation Panchkula in northern India during September–October 2016.


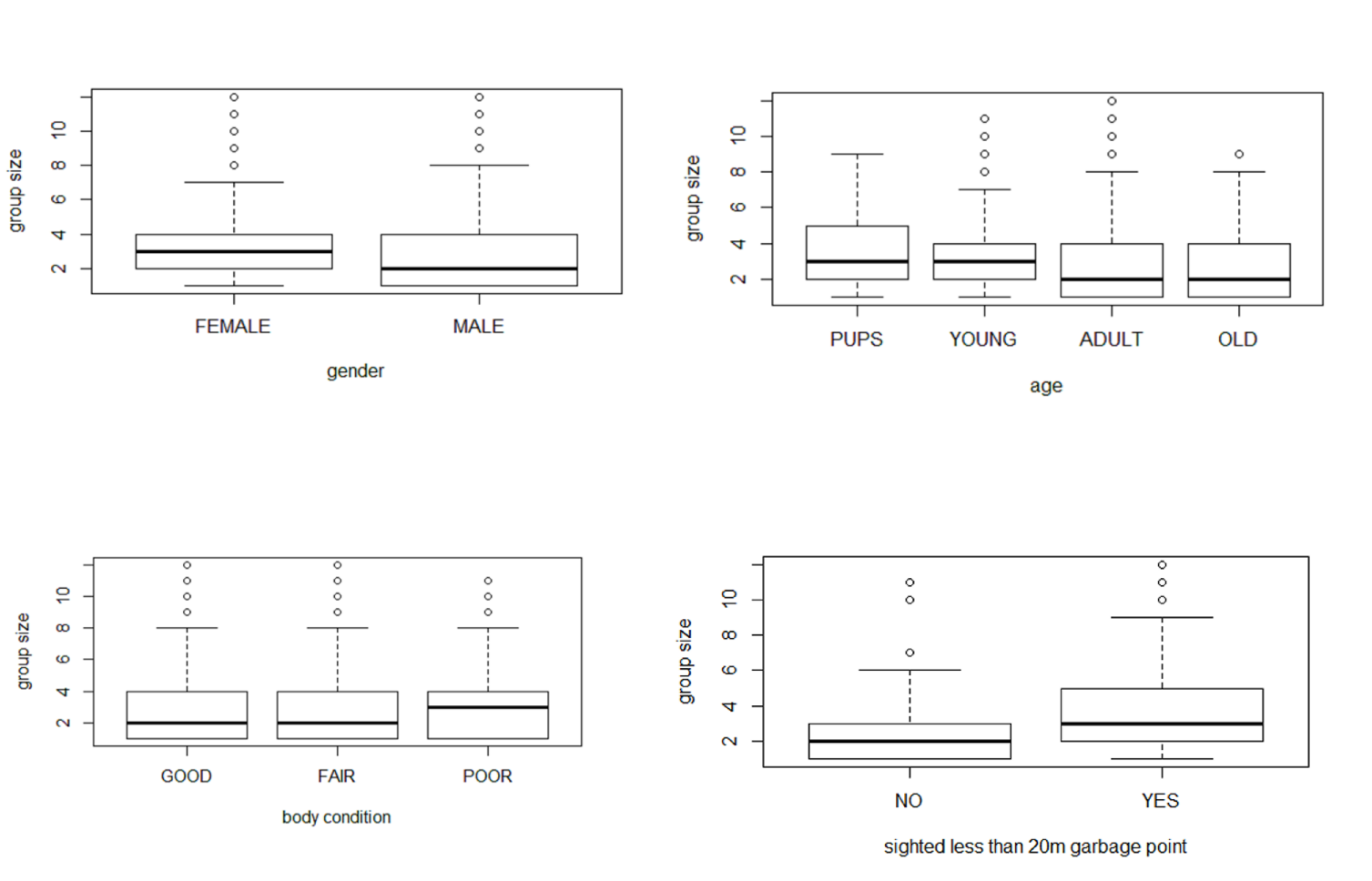


Supplementary Figure S3. Boxplot graphics for the univariable analyses of regression of the various extrinsic factors on the group size of FRD sighted during the enumeration survey in rural and urban setting.


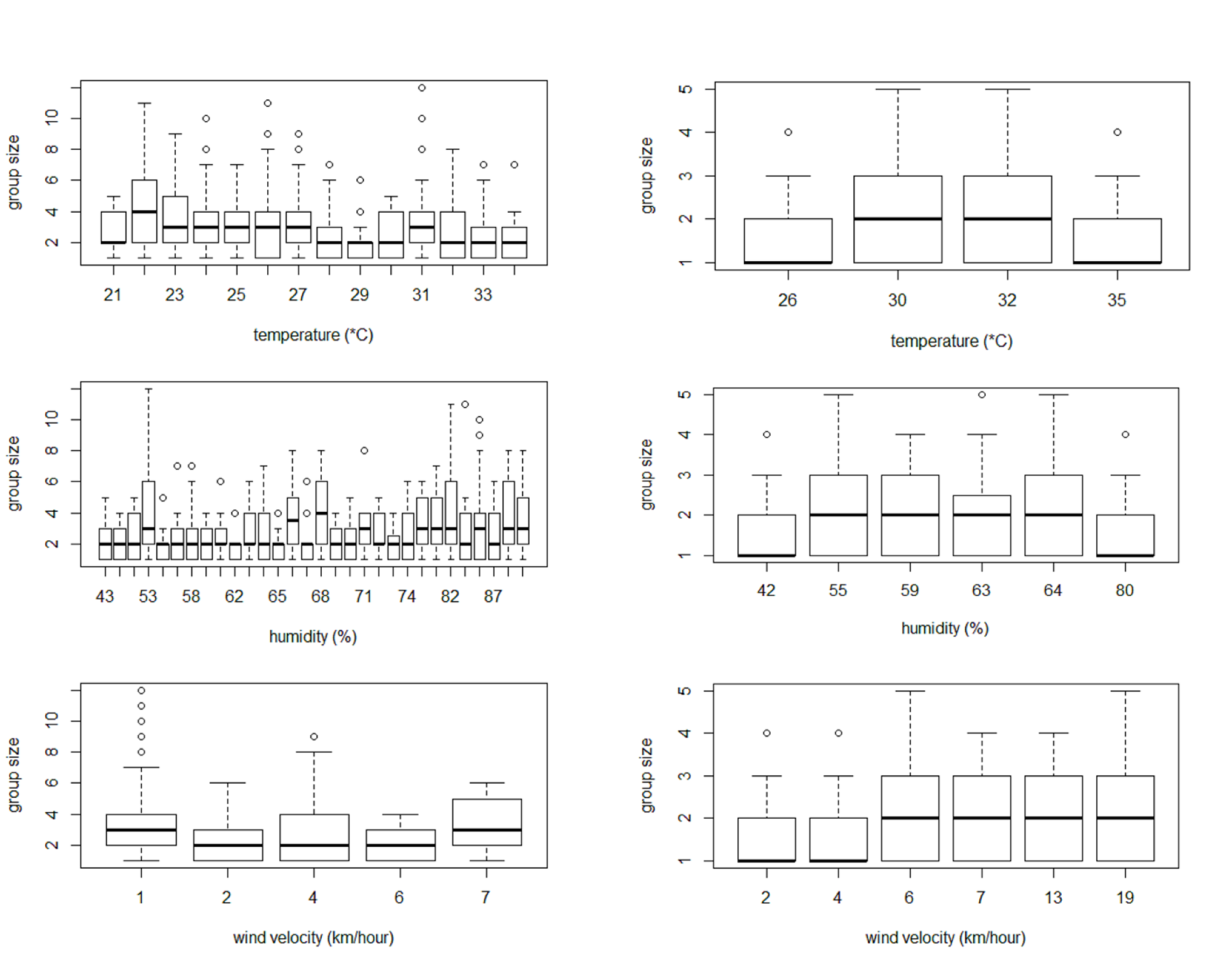


Urban Rural

Supplementary Figure S4. Boxplots for the univariable analyses of regression of resight probability on the group size of FRD sighted during the enumeration survey in rural and urban settings.

*
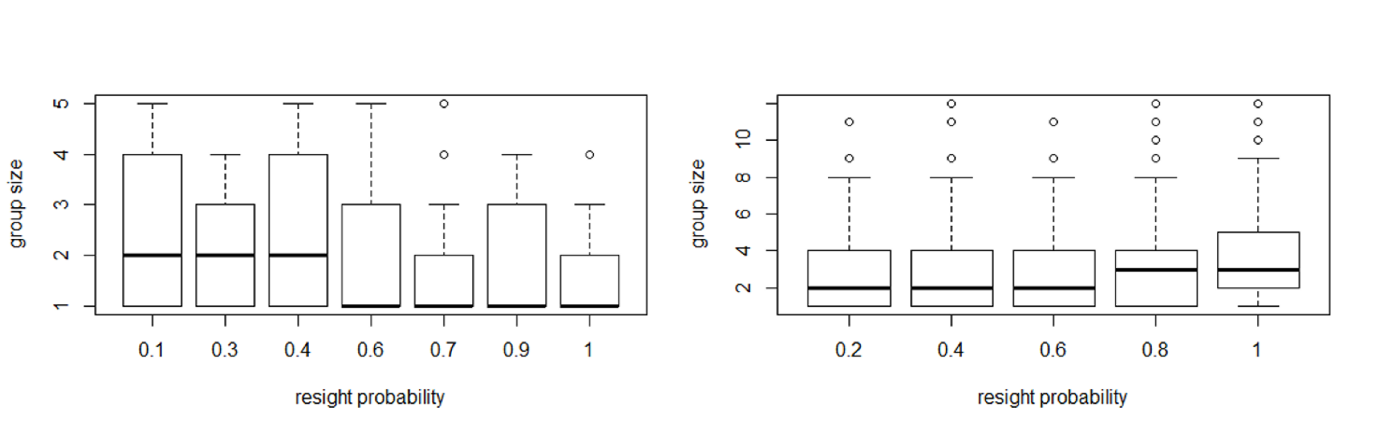
*

Supplementary Figure S5. Boxplots for the univariable analyses of de-sexed status probability on the group size of FRD sighted during the enumeration surveys in the urban setting in Panchkula.


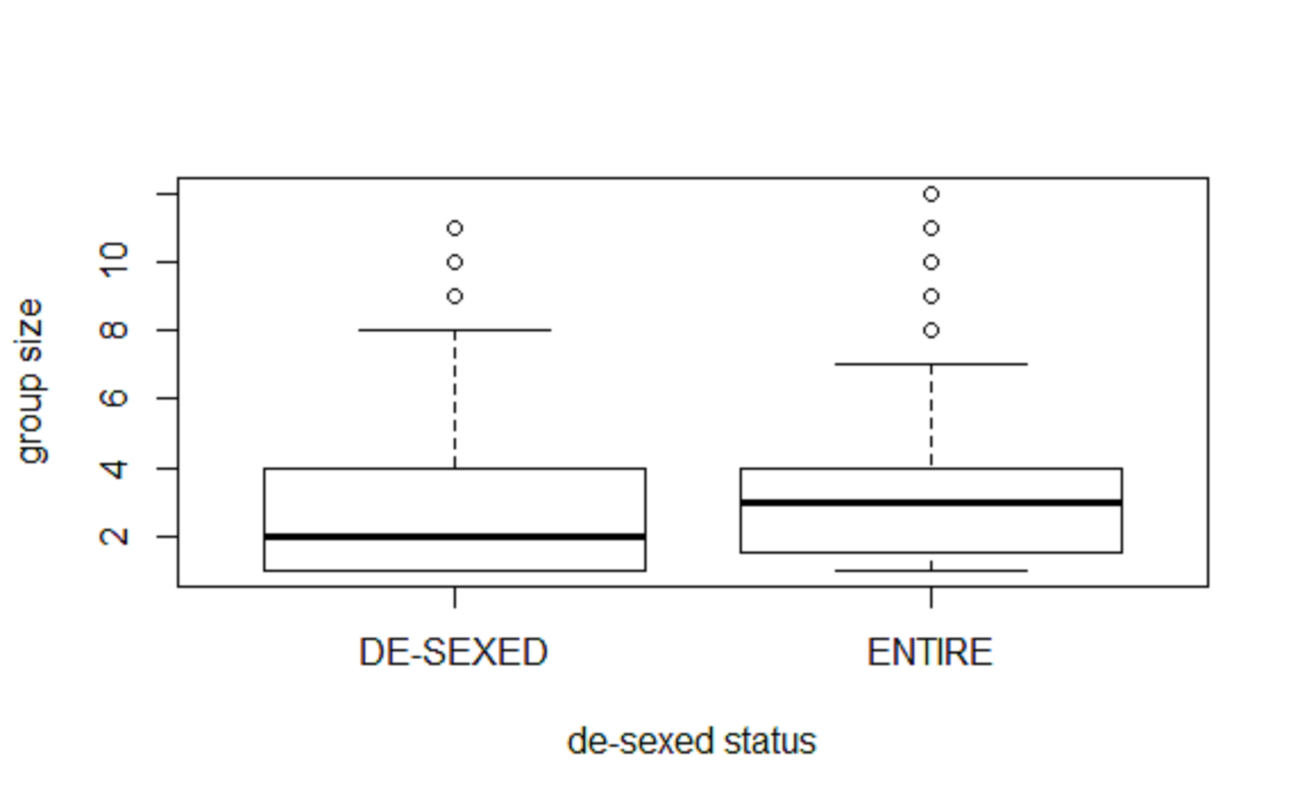


Supplementary Figure S6. Graphic representation of the odd ratios to compare the influence of various factors (probability of being sighted alone, gender, sighted within 20 m of garbage, if de-sexed, and body condition) on the home range of FRD sighted during the enumeration surveys in rural and urban settings through univariable analysis.


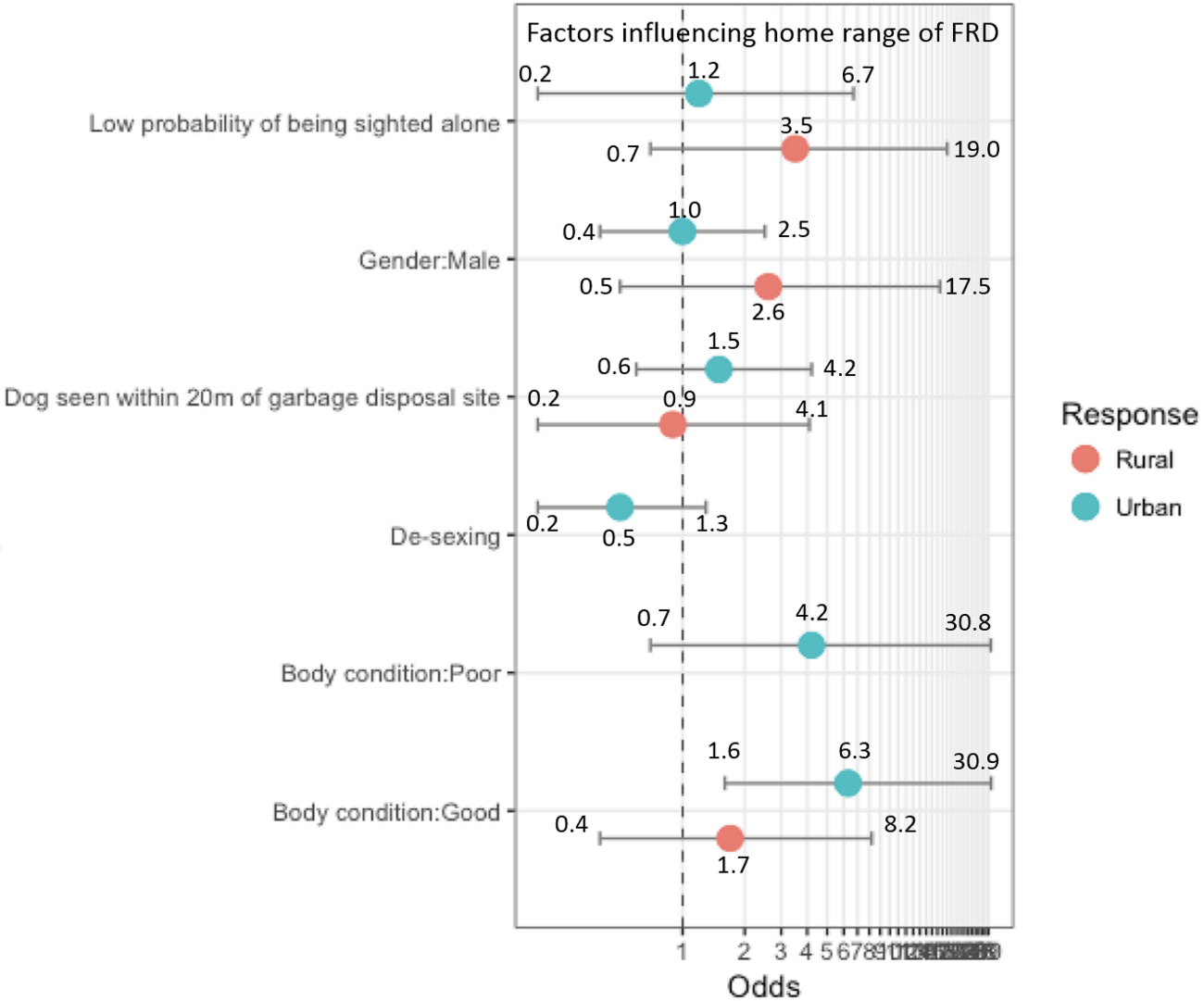

Supplement: Supplementary file 1 [file vaccines-07-00136-s001.docx]
